# Navigating uncharted waters: Challenges and opportunities

**DOI:** 10.4102/jcmsa.v1i1.14

**Published:** 2023-10-17

**Authors:** Soraya Seedat

**Affiliations:** 1 Department of Psychiatry, Faculty of Medicine and Health Sciences, Stellenbosch University, Cape Town, South Africa

‘*Change your opinions, keep to your principles; change your leaves, keep intact your roots*’.

*Victor Hugo*

The launch of the *Journal of the Colleges of Medicine of South Africa* (JCMSA) is one of the most exciting recent initiatives of the Colleges of Medicine of South Africa (CMSA).

This is at a time when the landscape of medical publishing is undergoing a rapid and remarkable transformation. This transformation is driven in large part by disruptive influences that straddle rapidly advancing technologies (including artificial intelligence [AI]), changing reader preferences, changing models of peer review, shifting attitudes towards preprints as currency to shore up trust in journal publishing, a ‘naming and shaming’ culture in respect of predatory publishers and fraudulent authors, and a flourishing ‘pay to publish’ culture that favours publishers over authors.^1^

Starting a new journal, under the umbrella of the esteemed CMSA, presents unprecedented opportunities. Several hurdles will need to be overcome, including the ‘holy grail’ of journal accreditation (i.e., the South African Department of Higher Education and Training [DHET] subsidy system), indexing (e.g., in PubMed Central), and obtaining a journal impact factor for the journal.

In the first editorial, I delve into the most pressing issues in this nascent phase and consider strategies to address these challenges, while harnessing the potential for growth and real impact.

## Quality versus quantity: Maintaining rigour in an era of prolific publishing

One of the most significant dilemmas faced by any new journal is maintaining a delicate balance between quality and quantity, while maintaining volume over time. The digital age has ushered in an era of prolific publishing, with an abundance of open access platforms and predatory journals vying for attention. Amid this deluge of information and choice, ensuring the integrity, reliability and rigour of scientific content in JCMSA is paramount. A new journal must consistently ensure rigorous peer-review processes, transparent editorial policies and ethical publishing practices and navigate the delicate balance of quantity over quality. *The Journal of the Colleges of Medicine of South Africa* will need to uphold publishing the best papers it receives and publishing them quickly. First steps would be growing the number of submissions, by inviting eminent clinicians and scientists in their field and members of the editorial board to submit articles (e.g., ‘hot’ topics, review articles) and in the near future publishing special (themed) issues. Although building reputation and quality will take time, attracting high-quality articles from the outset and fostering a reputation for high-quality content, JCMSA can distinguish itself from the sea of mediocre journals and garner the trust of researchers, clinicians and non-clinician readers alike.

## Ethical considerations: Navigating the complex terrain of publication ethics

Ethical considerations in medical publishing are multifaceted and extend beyond plagiarism and data fabrication. New journals also have to grapple with issues such as authorship disputes, conflicts of interest, and adherence to reporting guidelines. Striving for transparency and accountability is imperative in building credibility within the medical and dental community, and JCMSA is ably supported in this regard by its publisher, AOSIS. It does so by adhering to international guidelines and collaborating with recognised bodies like the Committee on Publication Ethics (COPE)^2^ for valuable guidance in establishing robust ethical frameworks, and ensuring that the publication process aligns with the highest standards of integrity.

## Diversity and inclusion: Representing the South African, African and global medical and dental community

The need for diversity and inclusion in medical and dental publishing cannot be overstated. The *Journal of the Colleges of Medicine of South Africa* intends making a concerted effort to represent the global medical and dental community, encompassing various geographical regions, ethnicities, genders and research domains, with an emphasis on highlighting South African and African research. Its wide interdisciplinary focus seeks to join disciplinary fault lines that currently exist, and encourages submissions across the board, from registrar trainees to established clinician scientists. By fostering an inclusive environment, JCMSA can enrich the content and perspectives that it publishes. Furthermore, the editorial team spans more than 20 constituent colleges, while editorial board members straddle geographical and disciplinary boundaries and are renowned in their fields. Their collective task will be to increase the visibility of JCMSA on an ongoing basis and, with the assistance of the publisher, promote the journal through their own submissions, disseminate content among colleagues, collaborators, trainees and students, and distribute promotional material at local and international conferences, workshops and other scientific meetings.

## Open access and sustainability: Navigating the financial conundrum

The open access movement has democratised access to scientific knowledge, but it has also raised concerns about financial sustainability. The *Journal of the Colleges of Medicine of South Africa* is immensely proud of its open access status where, in addition to being open access, article processing charges (APCs) will be covered by donations and sponsorships made to the CMSA for the journal. This is truly inspiring, especially as new medical and dental journals often face the dilemma of balancing open access with the need to cover operational costs, thereby levying APCs on authors to offset these costs. Exploring innovative funding models for JCMSA, such as consortium partnerships, philanthropic contributions, and collaborations with institutions, will be needed to offset the costs and secure a healthy financial status for the journal. In an accompanying editorial, Prof. Sam Nightingale, an editorial board member, addresses how and why we should revolutionise existing models of scientific publication. Embracing open access principles while adopting an assortment of strategies for long-term sustainability will be pivotal in ensuring that JCMSA can survive and continue to disseminate valuable medical and dental research.

## Technological advancements: Seizing opportunities for enhanced engagement

In the digital age, leveraging technology is indispensable for new medical publications aiming to enhance engagement and reach a wider audience. Incorporating multimedia elements (such as author video interview inserts on high-impact topics), and interactive figures can transform the reading experience and facilitate comprehension of complex medical and dental findings. Additionally, utilising social media platforms and personalised content recommendations will help JCMSA amplify the visibility of articles, can foster meaningful discussions, and spur new collaborations. Embracing technological advancements, including assistive AI software, will not only streamline the publication process but will also enhance the overall impact of the journal. AOSIS, in partnership with all editors of journals in its stable, is developing an AI policy to guide authors, reviewers and the editorial team.

## Data sharing and reproducibility: Fostering a culture of scientific integrity

Ensuring the reproducibility of research findings is the cornerstone of scientific integrity. As a new journal, JCMSA has a unique opportunity to advocate for transparent data sharing and robust methodologies. Encouraging authors to provide comprehensive datasets and detailed protocols can enhance the credibility of their published work. Furthermore, facilitating further post-publication peer review and replication studies will assist with validation, especially in local and regional contexts, and serve to foster a culture of trust and collaboration.

## Conclusion

We are embarking on a journey and traversing a dynamic landscape that will likely be fraught with challenges but is also ripe with opportunities. By upholding the principles of quality, ethics, diversity and accessibility, we can establish JCMSA as a reputable scientific outlet for highly innovative and contextually relevant medical and dental research. Navigating the complexities of this digital age will require an unwavering commitment to scientific integrity, innovation and adaptability.

By addressing the issues outlined here and embracing emerging global trends, I am hopeful that JCMSA can chart a course towards meaningful impact and contribute to advancing medical and dental research for the betterment of healthcare in the country and beyond.
